# Conservative Management of Atypical Endometrial Hyperplasia and Early Endometrial Cancer in Childbearing Age Women

**DOI:** 10.3390/medicina58091256

**Published:** 2022-09-11

**Authors:** Stefano Uccella, Pier Carlo Zorzato, Susan Dababou, Mariachiara Bosco, Marco Torella, Andrea Braga, Matteo Frigerio, Barbara Gardella, Stefano Cianci, Antonio Simone Laganà, Massimo Piergiuseppe Franchi, Simone Garzon

**Affiliations:** 1 Department of Obstetrics and Gynecology, AOUI Verona, University of Verona, 37126 Verona, Italy; 2 Obstetrics and Gynecology Unit, Department of Woman, Child and General and Specialized Surgery, University of Campania “Luigi Vanvitelli”, 80138 Naples, Italy; 3 Department of Obstetrics and Gynecology, EOC—Beata Vergine Hospital, 6850 Mendrisio, Switzerland; 4 Gynecology Department, San Gerardo Hospital, Milano Bicocca University, ASST Monza, 20900 Monza, Italy; 5 Department of Obstetrics and Gynecology, Fondazione IRCCS Policlinico San Matteo, 27100 Pavia, Italy; 6 Unit of Gynecology and Obstetrics, Department of Human Pathology of Adult and Childhood “G. Barresi”, University of Messina, 98121 Messina, Italy; 7 Unit of Gynecologic Oncology, Department of Health Promotion, Mother and Child Care, Internal Medicine and Medical Specialties (PROMISE), ARNAS “Civico-Di Cristina-Benfratelli” Hospital, University of Palermo, 90127 Palermo, Italy

**Keywords:** endometrial cancer, endometrial atypical hyperplasia, fertility-sparing treatment, conservative treatment

## Abstract

Total hysterectomy and bilateral adnexectomy is the standard treatment for atypical endometrial hyperplasia and early-stage endometrial cancer. However, the recommended surgical treatment precludes future pregnancy when these conditions are diagnosed in women in their fertile age. In these patients, fertility-sparing treatment may be feasible if the desire for childbearing is consistent and specific conditions are present. This review summarizes the available evidence on fertility-sparing management for atypical endometrial hyperplasia and early-stage endometrial cancer. Historically, oral progestins have been the mainstay of conservative management for atypical endometrial hyperplasia and stage IA endometrioid endometrial cancer with no myometrial invasion, although there is no consensus on dosage and treatment length. Intrauterine progestin therapy has proved a valid alternative option when oral progestins are not tolerated. GnRH analogs, metformin, and hysteroscopic resection in combination with progestins appear to increase the overall efficacy of the treatment. After a complete response, conception is recommended; alternatively, maintenance therapy with strict follow-up has been proposed to decrease recurrence. The risk of disease progression is not negligible, and clinicians should not overlook the risk of hereditary forms of the disease in young patients, in particular, Lynch syndrome. Hysterectomy is performed once the desire for childbearing desire has been established. The conservative management of atypical endometrial hyperplasia and early-stage endometrial cancer is feasible, provided a strong desire for childbearing and permitting clinical–pathological conditions. However, patients must be aware of the need for a strict follow-up and the risk of progression with a possible consequent worsening of the prognosis. More homogenous and well-designed studies are necessary to standardize and identify the best treatment and follow-up protocols.

## 1. Introduction

Endometrial cancer (EC) is the most common gynecologic malignancy in developed countries, being the fourth leading cause of cancer and the sixth cause of death due to cancer among women [1,2,3]. Although EC usually occurs after menopause, up to 14% of diagnoses are in premenopausal women [3], with over 5% of cancers diagnosed between 35 and 44 years, and 2% between 20 and 24 years [4]. Several risk factors are linked to the development of endometrial cancer, among them: high BMI or obesity, hyperinsulinemia, diabetes, hypertension, nulliparity, and anovulatory cycles [5,6].

EC is mainly diagnosed at an early stage with cancer confined to the uterus, as women seek medical attention for early symptoms, such as abnormal vaginal bleeding. In these cases, surgery is the standard treatment and consists of total hysterectomy (abdominal, laparoscopic, or robotic), bilateral salpingo-oophorectomy, and lymph node assessment, which, in the last decade, has evolved from a systematic lymph node dissection (LND) to the sentinel lymph node (SLN) mapping, raising new clinical questions, such as the management of low-volume lymphatic metastases [7,8,9,10,11]. Indeed, after surgery, subsequent adjuvant therapy might be indicated based on definitive pathology [12,13,14,15].

Nevertheless, although surgery alone is highly effective in low-risk EC, such as FIGO stage IA, endometroid, and grade 1–2 EC, with an overall survival rate of over 95% [16], total hysterectomy implies sterility in those cases diagnosed in premenopausal women. In these patients with the desire for childbearing, fertility-sparing management consisting of uterine and adnexa preservation has been investigated and proposed for both early-stage low-grade endometrioid EC and precursor lesions (atypical endometrial hyperplasia, AEH) [16].

Fertility-sparing approaches in young patients with a diagnosis of cancer are recommended by international guidelines [17]. However, the conservative management of uterus and adnexa preservation in women affected by gynecological malignancies, such as EC and AEH, has the risk of cancer persistence and recurrence [18,19]. Therefore, developing standardized and evidence-based management is paramount, with a clear definition of eligible criteria, primary lesion treatment, and follow-up modality. Different treatment strategies and follow-up protocols have been proposed and investigated, with progestins representing the main option [1,7,16,20,21]. Although the conservative approach in early-stage low-grade endometrioid EC and precursor lesions have become accepted [22], recommendations are mainly based on expert opinion or consensus [20,23]. The available evidence is heterogeneous and based on retrospective studies with different treatment and follow-up protocols. In this narrative review, we summarize fertility-sparing treatment in premenopausal women with the desire for childbearing. We aimed to provide an overview of treatments and associated oncological and reproductive outcomes. Appropriate knowledge of available evidence will help clinicians to better counsel and manage patients with a diagnosis of EC or AEH desiring the preservation of their fertility.

## 2. Work-Up for Fertility-Sparing Treatment

### 2.1. Patient’s Eligibility Criteria

The first step is to perform an initial assessment of the patient through a complete anamnestic and family history work-up, and clinical examination [23].

Then, a proper diagnosis and staging of the disease with the exclusion of myometrial invasion and distant metastases [24,25,26] through imaging, such as a CT scan, enhanced magnetic resonance imaging (MRI), or expert ultrasound examination, is mandatory. The adequate collection and review of the histological sample by an expert pathologist is also fundamental [21,23,27,28]. No universal sampling method has yet been defined; however, hysteroscopy with endometrial biopsy is recommended over dilation and curettage [23]. The appropriate initial evaluation must confirm the eligibility for fertility-sparing management: well-differentiated endometrioid EC or AEH, disease limited to the endometrium, no contraindications to medical therapy, and strict adherence to follow-up [22].

### 2.2. Lynch Syndrome and Association with Concomitant Tumors

Lynch syndrome is an inherited condition predisposing to colorectal cancer, EC, gastric cancer, and other malignancies, and accounts for 2 to 5% of all endometrial malignancies [29,30,31]. This autosomal dominant hereditary syndrome with high penetrance is caused by the mutation of a mismatch repair gene that is involved in DNA mismatch repair (MMR)/microsatellite instability (MSI): MLH1, MSH 2 or 6, and PMS2 [6]. It affects 1 out of 370 to 2000 persons in Western countries [32,33]. The risk of developing an endometrial malignancy is approximately 50% during the lifespan, and typically, affected women are younger than sporadic EC patients [34]. For these reasons, clinicians should collect a detailed family history before considering patients eligible for conservative treatment, and in case of any suspicion, genetic testing should be promptly obtained. To identify patients with Lynch syndrome, MMR status/MSI testing is recommended in all endometrial carcinoma samples [23,35]. Another important aspect is that women affected by superficially invasive EC under the age of 50 have a high risk of having a synchronous initial, asymptomatic ovarian cancer if they have a positive family history of ovarian or breast cancer [36]. It is paramount to provide adequate screening and counseling to these women, in order to identify patients in whom conservative treatment should not be offered. Based on the ultimate ESGO recommendation, prophylactic hysterectomy and bilateral salpingo-oophorectomy should be performed at the competition of childbearing and ideally before the age of 40 years [23].

### 2.3. Biomolecular and Genetic Prognostic Factors in Endometrial Cancer

In 2013, the Cancer Genome Atlas Research Network published a molecular diagnostic classification that defines four prognostic categories in endometrial cancer: POLE ultra-mutated, microsatellite instability hypermutated, low copy-number tumor, and high copy-number tumor [6,23,37].

These groups include different molecular and genetic factors that play a role in the physiology and pathology of the disease, influencing the prognosis. Hence, they are used for the risk stratification of patients. Some mutations are already well-known and considered in the clinical setting for the management of patients; however, they have not yet been included in the decision algorithm for fertility-sparing treatment [6,38]. A recent review [38] identifies and summarizes the currently established genetic prognostic factors included in the prognostic categories, aiming to establish whether they facilitate the decision for fertility sparing in early endometrial cancer. The authors conclude that the data are still inconsistent and not universal. However, based on recurrence rate, risk of metastases, and the mortality of early endometrial cancer, they defined PTEN and POLE alteration to be good prognostic factors favoring fertility-sparing treatment. On the other hand, poor prognostic factors were found to be PIK3CA, HER2, ARID1A, P53, L1CAM, and FGFR2. However, for decision making, the patients’ clinicopathological characteristics still have to be taken into consideration [38]. Larger clinical trials are deemed necessary to identify the role of these prognostic factors in the treatment algorithm of these tumors.

## 3. Fertility-Sparing Treatments for Endometrial Cancer

### 3.1. Oral Progestins

Systemic hormone therapy based on oral progestins is historically the main fertility-sparing treatment for early-stage low-grade endometrioid EC and AEH [39,40]. Their use is based on the pathogenesis linked to excessive estrogen exposure without the counterbalanced effect of progesterone [5,41]. Progestins act on EC cells, causing growth suppression, the downregulation of estrogen receptors, and the activation of enzymes that are involved in estrogen metabolism. Progestin interferes with cell cycle regulation processes, such as those regulated by cyclin-dependent kinase (Cdk), reinforce p27 expression, a cyclin E-Cdk2 complex inhibitor, with the suppression of the cell cycle. Progestins are also able to promote cellular secretory differentiation, inhibit inflammation and invasion, and have an anti-angiogenic effect [42]. The most investigated progestins are medroxyprogesterone acetate (MPA, 20–1500 mg/day) and megestrol acetate (MA, 40–480 mg/day), alone or in combination with other treatments [16,43]. MPA and MA are progesterone agonists with antiandrogenic and antiestrogenic effects [44]; both are orally administered, although MPA can also be used intra-muscularly. Other progestins are hydroxyprogesterone caproate, norethisterone acetate, natural progesterone, dydrogesterone, and oral contraceptive pills [43,45]. Commonly reported adverse events are weight change (gain more common than loss), transient liver dysfunction, coagulation abnormality, and breast pain [43,46]. No instances of stroke, deep vein thrombosis, or other life-threatening complications have been reported [43]. Regardless, progestins are the most investigated fertility-sparing treatment for endometrioid EC and AEH, but there is no consensus on the optimal dosage and duration of therapy. Several protocols have been investigated, with dosages ranging between 20 and 1500 mg/day for MPA and 40–480 mg/day for MA [5,16]. In this regard, although randomized controlled trials investigating the proper dosage of progestins are not available, evidence that high-dose progestins are more effective than low-dose regimens has been reported [43,47]. A recent case series with patients receiving either low dose (MA 80 mg daily; MPA 150 mg) or high dose progestins (MA ≥ 160 mg; MPA 500 mg ×2/week) reported complete remission in a low- and high-dose regimen of 55.6% and 73.3%, respectively (Table 1) [43]. Consistently, a retrospective study testing MA 80 mg and MPA 100 mg achieved a complete response of 55%, lower than those reported in the literature (71–85%) (Table 1). However, a meta-analysis of 28 studies and 1038 patients did not confirm a relationship between the dose and response to treatment [48], although the recently published ESGO guidelines support high dosage regimens, suggesting MPA at the dose of 400–600 mg/day or MA at the dose of 160–320 mg/day [23]. The duration of treatment is also very heterogeneous among studies. The progestin effect might be evident on endometrial cells as early as 10 weeks after treatment begins; however, most studies wait at least 3 months for AEH and 6 months for EC before assessing the treatment response [7]. The recommendation from the ESGO guidelines is to proceed with surgery if no response is observed after six months of treatment. Nevertheless, the minimum treatment length before concluding for treatment failure and moving forward with surgery is still debated. The metanalysis by Koskas et al. determined the remission probability based on treatment length (Table 1) and observed a plateau in the complete response rate (approximately 80%) after only 12 months, suggesting that radical surgery should be considered if no remission is obtained within this time frame [44]. A further retrospective study on long-term fertility-sparing treatment (FST) including 122 patients with EC [49] suggests 15 months as the cutoff for optimal conservative treatment. A treatment period of up to 18 months was proposed by Wang et al., who observed in their retrospective study on 67 patients that the cumulative complete response rates progressively increased at 6, 9, and 18 months to 58%, 76%, and 95.5%, respectively, without affecting recurrence rate or pregnancy rate [16,50,51,52,53]. The duration of treatment might be influenced by the patient’s characteristics as well, such as body mass index (BMI) and insulin resistance [54]. Notably, the reported likelihood of resolution for obese women (BMI ≥ 35) at 12 and 18 months was, respectively, 33% and 66% [16,55]. Complete response, recurrence, pregnancy, and live birth rates after progesterone therapy extrapolated from the main available metanalyses are listed in Table 1. The recent metanalyses by Zhao [56], including 446 patients with well-differentiated EC and younger than 40 years, reported a pooled complete response rate with oral progestins of 82% (95% CI: 74–92%). The pooled relapse and pregnancy rate were 38% (95% CI: 31–45%) and 70% (95% CI: 62–79%, *p* = 0.68), respectively, and the live birth rate was 63% (95% CI: 55–73%). A similar rate was observed by Zhang et al. [57] in a meta-analysis including up to 54 studies and comparing different treatments. Women with EC treated with oral progestogens alone had a pooled regression rate of 79.47% (95% [CI]: 73.19–85.10), whereas a higher regression rate of 88.74% (95% [CI]: 81.70–94.25) was observed in women with AEH. Recurrence rate and pregnancy rate were 27.34% (95% [CI], 18.19–37.56) and 32.28% (95% [CI], 22.87–42.48) for EC and 9.20% (95% [CI], 3.91–16.43) and 28.74% (95% [CI], 19.20–39.35) for AEH. Compared to those with EC, women with AEH achieved a statistically significant higher regression rate (*p* = 0.0417) and a relatively lower recurrence rate (*p* = 0.004), without any difference in live birth rates (*p* = 0.73) [57]. These results confirm the increased likelihood of a response to hormonal therapy of AEH than EC, which was observed earlier by Gunderson et al. [45]. Other factors associated with the complete response rate have been identified, such as the progestin itself [44,56,58]. Notably, although MPA is the most used treatment, MA has been associated with a higher complete response rate, which is potentially explained by its higher bioavailability [44,58]. Several recently published observational and retrospective studies additionally highlighted the negative effect of high BMI, older age, insulin resistance, and PCOS on the probability of complete response [16,47,54,59,60,61].

### 3.2. Levonorgestrel-Releasing Intrauterine System (IUS)

Levonorgestrel-releasing intrauterine systems (IUSs) provide a local higher dose concentration of progestins than the oral route, avoiding the adverse effects linked to the systemic administration of progestogens [46,48,64], with an expected increased compliance of the patient. Nevertheless, available evidence regarding the use of IUS as a fertility-sparing treatment for EC and AEH is heterogeneous, with a complete response rate ranging from 37.1% to 100% and an estimated median time to achieve a complete response of 9.8 months [16,64]. Table 2 displays the main studies on IUSs, which have been investigated alone and combined with gonadotropin-releasing hormone receptor agonists, progestins, hysteroscopic resection, metformin, and dilation and curettage. Westin et al. prospectively followed up 57 patients with IUS for 12 months and reported an overall complete response rate of 79% [65], which, consistently with studies on oral progestins, was higher for AEH than EC (91% vs. 54%). Pooled complete response and recurrence rates of 76% (95% CI: 67–83%) and 9% (95% CI: 5–17%) were reported by a metanalysis conducted on 1038 patients [48]. However, the relapse rate appeared to be too optimistic compared to most studies on IUSs [16,57]. Notably, a significantly higher overall recurrence rate of 41.5% was reported by a retrospective study on 48 patients treated with IUS [66], although the authors stressed that up to 64.7% of recurrences occurred after IUS removal. Recently, IUS for early EC and AEH in obese women was investigated by the FeMMe trial, an open-label, three-arms, randomized phase II clinical trial. Patients with a BMI > 30 Kg/m^2^ with early EC or EAH had a levonorgestrel-IUS inserted and were randomized into an observation, weight loss intervention, or oral metformin group [67]. Interestingly, the complete response rate at 6 months was 67%, 61%, and 57% for the weight intervention, observation, and oral metformin group, respectively [67]. Concerning reproductive outcomes, differences between oral progestins and IUS were not demonstrated [48]. Moreover, reproductive outcomes reported in the retrospective study by Maggiore et al. (73.7%) and Novikova et al. (68%) were satisfactory [66,68], although the overall live birth rate was 27% with spontaneous conception and 38% after assisted reproductive therapy [66,68]. A subgroup of studies investigating IUSs as a fertility-sparing treatment for EC and AEH focused on factors potentially predicting the response to the IUS. The absence of exogenous progesterone effect defined as small inactive glands and pseudodecidualized stroma in a biopsy performed after 3 months of treatment was associated with a lack of response after 12 months [65]. Other markers potentially guiding the transition to an alternative therapy due to their higher prevalence in non-responders were the high expression of Ki67 (a marker of cell proliferation) and low expression of DKK3 (tumor suppressor) at baseline [65]. Based on available evidence, the recently published ESGO guidelines recognized the satisfactory pregnancy rate and low recurrence rate associated with IUS when combined with gonadotropin-release hormone receptor agonist or progestin, allowing the possible use of IUS as a treatment for EC and AEH [23].

### 3.3. Metformin

Metformin is a lipophilic biguanide that exerts an insulin-sensitizing effect inhibiting hepatic gluconeogenesis and opposing glucagon action [16]. Metformin is the first-line therapy for type 2 diabetes mellitus, is readily available worldwide at a low cost, has a well-defined safety profile, and is prescribed for various non-diabetic conditions, such as PCOS, cardiovascular diseases, and obesity [71]. Among multiple effects, metformin has demonstrated anti-neoplastic properties for several malignancies (colorectal cancer, pancreatic cancer, and lung cancer) including EC [1], although the mechanism of action against EC and AEH is mainly unknown. First, metformin acts on well-known risk factors, such as type 2 diabetes mellitus and insulin resistance [72]. Second, metformin affects endometrial maturation, proliferation, and implantation processes through its modulatory effect on steroid receptors, as it decreases estrogen receptors and increases progesterone receptor expression [22,73]. Therefore, metformin appears to enhance the progesterone’s inhibitory effect on endometrial cell proliferation, potentially overcoming elements of progesterone resistance caused by the downregulation of progesterone receptor expression, which might positively impact the relapse rate [22,71]. Other effects are the inhibition of cancer stem cell-like subpopulation in cases of intraepithelial neoplasia and the prevention of the conversion of epithelial cells into mesenchymal cells. Interestingly, at a molecular level, metformin shares similar molecular targets with current drugs studied for advanced diseases, such as sorafenib and everolimus [71]. Table 3 summarizes the most recent studies on metformin for fertility sparing in EC and AEH. Mitsuhashi et al. published a phase 2 trial enrolling 38 patients who received MPA 400 mg, aspirin 100 mg, and metformin 750 mg/day (up to 2250 mg/day) for 24 weeks [74]. The complete response rate was 64% after 6 months and 81% after 9 months. Subsequently, metformin was extended until conception or disease recurrence. The recurrence rate was 10.3% after a median of 38 months, and the clinical pregnancy rate among those who sought conception was 50%. Long-term outcomes were reported in a second study [75]. The complete response rate achieved 97% within 18 months (100% in AEH/95% in EC), and the recurrence rate was 13.1% after a median follow-up time of 57 months, with a 5-year recurrence-free survival of 84.8%. Pregnancy and live birth rates were 61% and 45%, respectively. In this retrospective study, the authors compared the results with a control group, receiving only MPA, which had a complete response rate of 87% and a recurrence rate of 50%, suggesting a better prognosis with MPA plus metformin, although the prevalence of PCOS and average BMI were higher in the metformin plus MPA group [75]. Mitsuhashi et al. designed the FELICIA trial to identify the appropriate metformin dose to add to MPA for the fertility-sparing treatment in patients with EC or AEH. This randomized phase IIb dose-response trial will measure the three-year recurrence-free survival as a primary endpoint. Secondary endpoints are the overall response rate, pregnancy and live birth rate, toxicity, and changes in BMI and insulin resistance [76]. In contradiction with the evidence provided by the studies on MPA plus metformin, the metanalyses by Prodromidou et al. on MA plus metformin versus MA alone did not find any difference in either complete response or partial response rates [1]. However, the metanalysis included only two randomized controlled trials. The recent meta-analysis by Chae-Kim et al. including any study investing progesterone plus metformin versus progesterone alone found a lower relapse rate in patients receiving both progestins and metformin than in those patients who received only progestins. However, remission, clinical pregnancy, and live birth rates did not differ [22]. Similarly, the metanalysis of Cho et al. also confirmed a better outcome when metformin was added to fertility-sparing treatment in EC, prolonging the overall survival and reducing the risk of cancer relapse [71]. Conflicting results may rely on the type of progestin and the route of administration. In a recent retrospective study by Matsuo et al., women with AEH were divided into oral versus intrauterine progestins with or without metformin. The highest complete response rate was observed in the group that received IUS plus metformin. The authors hypothesized that observed differences may be caused by a direct interaction between metformin and oral progestins potentially altering the metformin metabolism. Moreover, oral progestogens may indirectly counteract the effect of metformin by causing weight gain and increasing the inflammatory cytokines that are involved in oncogenesis. Therefore, the authors concluded that in a predominant obese population, concurrent metformin may offer treatment benefits when used with IUS [77]. Even if diabetes mellitus does not seem to affect the outcome of the conservative treatment of EC and AEH, metformin appears to improve overall survival in patients with EC [23].

### 3.4. Other Drugs

In association with progestin therapy (local or systemic), different drugs have been investigated such as GnRH agonists, aromatase inhibitors [79], aspirin [80,81], and statins [82]. GnRH agonists combined with IUS have demonstrated satisfactory results in terms of complete response and recurrence rate. After one year of IUS combined with 6 months of GnRH analogs, up to 95% of AEH and 57% of EC achieved a complete response, with an overall recurrence rate of 20% and a pregnancy rate of 85% [16,83]. A complete response rate was achieved by all 24 patients by Pahov et al. [84]. This was also observed in a recent retrospective study evaluating different treatment protocols for the conservative management of EC and AEH [68] (Table 1). AEH and EC treated with IUS plus GnRHa and dilation and curettage had a complete response rate of 100% and 96%, respectively. Similarly, Zhou [85], in a retrospective study including 29 patients younger than 45 years who received GnRH analogs with either IUS or letrozole, had a complete response rate of 88% and 100% and a recurrence rate of 5.9% and 8.3% for AEH and EC, respectively. Letrozole is an aromatase inhibitor reducing estrogen levels by inhibiting estrogen synthesis in the ovaries and peripherical tissues. As a consequence, letrozole suppresses receptor-mediated tumor growth in hormone-sensitive tumors [85]. GnRH analog associated with letrozole is an interesting non-progestin-based treatment for the conservative management of EC and AEH, which may be suitable for those patients who do not tolerate progestogens due to serious adverse effects [16,85].

### 3.5. Hysteroscopic Resection

Hysteroscopy is the gold standard for the diagnosis and treatment of intracavitary pathologies of the uterus. In the case of EC and AEH, hysteroscopy allows the performance of a biopsy under the direct vision of the lesion, and it was associated with higher sensitivity and specificity than blind endometrial curettage [23,86]. Nevertheless, regarding the treatment of EC and AEH, Mazzon et al. were among the first to propose the use of hysteroscopy for the fertility-sparing management of EC and AEH in premenopausal women with childbearing desire. They proposed a three-step technique based on the hysteroscopic resection of the tumor (I. histopathological sample), the adjacent endometrium (II. Histopathological sample), and the underlying myometrium (3–4 mm in depth) [16,86,87], followed by hormone therapy. They reported a small case series of six young patients with EC that underwent hysteroscopic resection followed by MA 160 mg/day. All patients achieved complete responses. After a median follow-up time of 50.5 months, none of the women relapsed, and the live birth rate was 66%. Similar results were achieved by more recent studies with a similar treatment protocol [46,68,88,89,90,91,92,93]: the complete response rate with hysteroscopic resection ranged from 65% to 100%, with a pregnancy rate of 25–100% [86]. When comparing different fertility-sparing treatments for AEH and EC, several metanalyses and systematic reviews reporting different treatment strategies pooled similar results with hysteroscopic resection, usually combined with progestins or GnRHa, being superior to other treatments in terms of complete response and recurrence rate (Table 1) [7,46,56,58,68]. For instance, in a recent meta-analysis, hysteroscopic resection, IUS, and oral progestins were compared. The hysteroscopic resection group achieved a higher complete response rate (95% vs. 82% in oral progestins group and 69% in IUS group) and pregnancy rate (84% vs. 70% in oral progestins group and 48% in IUS group), and a lower recurrence rate (16% vs. 38% oral progestins group and 30% IUS group) [56]. The optimal results achieved after hysteroscopic resection were explained by the cytoreductive effect on the primary tumor that might increase the effectiveness of the progestin therapy [86]. Not surprisingly, ESGO recommends hysteroscopic biopsy for diagnosis and hysteroscopic resection before progestin therapy [23]. It is worth nothing that although hysteroscopic resection was associated with a higher rate of positive peritoneal cytology, this marker was not associated with prognosis [23].

## 4. Fertility-Sparing Treatment Follow-Up

Proper tailored follow-up is necessary for patients undergoing fertility-sparing treatment for endometrial hyperplasia or cancer. The current recommendation suggests hysteroscopic guided biopsy and imaging at 3–4 and 6 months. If no response is achieved after 6 months, surgery is recommended [23]. Partial responders may continue the treatment for another 3–6 months [5]. Consistently, in responders who desire to postpone pregnancy, the evidence suggests that maintenance therapy with progestins may decrease the risk of recurrence after complete response [23]. This was also observed in a small prospective observational study reporting no recurrence in women with maintenance therapy [64]. Prolonged progesterone exposure with IUS maintenance or progesterone treatment after childbirth was associated with decreased rates of recurrence in women with an initial diagnosis of AEH [68]. Strict surveillance with 6 months ultrasound and clinical follow-up is recommended, with hysteroscopy and endometrial biopsy suggested only in the case of abnormal vaginal bleeding or atypical ultrasound findings [23]. An evaluation at a fertility clinic before and after conservative treatment is advised, in order to help these women to fulfill their desire to be pregnant, with surgery recommended soon after childbearing [23].

## 5. Current and Future Prospective of Fertility-Sparing Treatments

Conservative treatment for AEH and EC with uterine and adnexa preservation in premenopausal women with the desire for childbearing has reached satisfying results in terms of complete response, recurrence, pregnancy, and live birth rates. However, the available evidence is heterogeneous and presents many limitations. Only a few studies are randomized controlled or non-randomized trials. Most pieces of evidence are based on retrospective observational designs, which introduce possible confounders and selection biases. Moreover, the fertility-sparing treatments differ between studies, even between studies investigating the same medication. Progestins, although being the main treatment option, have been investigated with multiple regimens, representing one of the multiple reasons explaining disagreements in provided results. Other factors, such as the follow-up length and women’s characteristics, appear to affect fertility-sparing outcomes. The complete response rate varies based on fertility-sparing treatment, the length of follow-up, and patient BMI, and available studies differ in many of these criteria. On that basis, a standardized evidence-based primary treatment and follow-up modality are still not defined. Future research may benefit from a clear definition of treatment regimes, minimum follow-up length, and patient eligible criteria. Thereafter, appropriate study designs, favoring prospective trials, and appropriate sample sizes must be implemented.

In summary, extensive work has been carried out to provide a treatment option for premenopausal women with the desire for childbearing to conserve their fertility until their desire for pregnancy is fulfilled. However, primary surgery based on hysterectomy and bilateral adnexectomy is still strongly recommended by the ESGO guidelines as the first-line therapy [23]. Indeed, when counseling the patients, it is paramount to provide extensive data on the complete response and recurrence rate of fertility-sparing treatments, highlighting that the standard of care remains hysterectomy and bilateral adnexectomy. Any fertility-sparing treatment may potentially have worse oncologic outcomes due to disease persistence or relapse, even related to a less accurate staging by imaging than by uterine pathology after hysterectomy. Hence, adherence to a close follow-up is mandatory [20,46].

Nowadays, there is no unique fertility-sparing protocol for the management of EC or AEH. Oral progestins are the most common conservative treatment with a consistent body of evidence. The satisfactory response rate is counterbalanced by the high recurrence rate, and the systemic effect of progestogens may decrease the patients’ compliance. The intrauterine progestin therapy with IUSs helps to reduce adverse events of systemic progesterone therapy and combined with oral progestins, GnRH agonists, or metformin can achieve a satisfactory pregnancy rate and low recurrence rate [23]. In the absence of randomized trials, limited evidence suggests that hysteroscopic resection before progestin therapy (local or systemic) with or without GnRH agonist may provide an additional benefit [23]. Therefore, hysteroscopy allows both a more accurate diagnosis with the collection of a proper tissue sample for biopsy and represents an interesting option to increase the efficacy of hormone therapies. The only concern is the risk of peritoneal dissemination of malignant cells through retrograde flow caused by liquid distension of the uterine cavity [16,86]. However, there is no available evidence of a worse prognosis associated with hysteroscopy, and published studies have demonstrated its feasibility and safety [86].

Regardless of the chosen fertility-sparing treatment, close follow-up is mandatory for all patients managed conservatively. Indeed, the probability of recurrence never reaches a plateau, and prompt identification of the disease is paramount for prognosis [44]. In this regard, the use of maintenance therapy with progesterone or pregnancy after the complete response has proven to reduce the relapse rate [68,94].

It is worth noting that recurrence is highly detrimental to the establishment of pregnancy, with patients experiencing relapse found to be 80% less likely to conceive [95]. A recent retrospective study on 68 patients evaluating pregnancy-associated factors after fertility-sparing therapy observed a lower relapse rate in the pregnancy group compared to the non-pregnancy group—16.7% and 40.6% [95]. The study identified normal BMI, a shorter time to complete response, a prolonged three-month treatment, fewer hysteroscopy procedures, and a thicker endometrium, as positive indicators for successful pregnancies [95].

Pregnancy after complete remission can be spontaneous or with assisted reproductive technology, although the latter can increase the chance of pregnancy significantly [46]. Even if there is concern about the possible negative effect of fertility drugs on EC progression or recurrence, no significant difference in recurrence rate between cases with assisted reproductive technology treatment and those with spontaneous pregnancy was found [95]. In the pregnancy group, 78.7% of pregnancies were achieved by assisted reproductive technology treatment, with a live birth rate of 53.2% [95]. If we consider that the probability of recurrence never reaches a plateau [44], with the need for strict follow-up and maintenance treatment until pregnancy is achieved, assisted reproductive technology should be proposed to infertile women as soon as possible after the complete response is achieved.

Finally, after completion of childbearing, in case of non-response to hormonal therapy, or in case of recurrence, hysterectomy is recommended [23]. Notably, only in highly selected cases under strict surveillance can conservative treatment be repeated [23]. In this regard, some studies demonstrated good oncologic outcomes and pregnancy rates after the conservative treatment of relapse cases [16,51,94,96,97], with a complete response rate after re-treatment up to 96.4% and 98.1% for AEH and EC [97]. However, caution is required as limited evidence is available, and a worse recurrence rate and lower 5-year recurrence-free survival have been demonstrated [97]. In the future, more homogenous and better designed, possibly multicenter, studies are warranted to standardize eligible criteria, primary lesion treatment, and follow-up modality for the conservative fertility-sparing management of AEH and EC [6].

## 6. Conclusions

Fertility-sparing treatment for EC and AEH is a possible therapeutic option for premenopausal women desiring to preserve their fertility. Satisfactory oncologic outcomes and pregnancy rates have been achieved through conservative management. Current evidence would suggest that in carefully selected patients with low-stage EC or AEH, the use of fertility-sparing options, especially oral progestins, can achieve a complete response in up to 82% [56] and 88% for EC and AEH, respectively [57]. For complete responders who try to conceive a pregnancy, up to 63% will achieve a livebirth [56]. Nevertheless, stressing that in complete responders up to 38% will develop a relapse is paramount [56].

Progestins, local or systemic, are the most investigated and recommended therapeutic option. The efficacy of oral progestins appears to increase when combined with metformin, GnRH analogs, or hysteroscopic resection. The latter has proven to be effective and is associated with a higher complete response, up to 95% [56], and live birth rate, up to 72% [56], with a lower relapse rate, up to 16% [56]. Nevertheless, the best treatment protocol in terms of progestin type, dosage, and duration is still not defined. Most pieces of evidence are from the metanalyses of heterogenous retrospective and prospective observational studies. Therefore, randomized controlled trials and more homogenously designed prospective studies are deemed necessary to better define the population, treatment, and follow-up protocols. The potential of biomolecular and genetic prognostic factors will help to identify who will benefit the most and suffer the lowest risk from conservative treatments.

Proper counseling for the patients is mandatory. Clinicians should stress that evidence regarding effectiveness and safety is limited, and that conservative treatment is not the standard and recommended treatment for EC and AEH. The patient must be aware that adherence to strict surveillance is deemed necessary until pregnancy is achieved to detect and promptly treat any recurrence, and that recurrence or inappropriate initial staging may significantly worsen the prognosis.

## Figures and Tables

**Table 1:** Main metanalysis/systemic reviews on progestin therapy for endometrial cancer and atypical endometrial hyperplasia.

Study	Type of Study	Pt (*n*)	Treatment (% or *n*)	Complete Response	Relapse	Pregnancy	Live Birth (% or *n*)
Baker, 2011 [62]	Metanalysis	219 prog:	Oral Prog:	Oral Prog	Oral Prog 20.1%	Oral Prog 43%	Oral Prog
		117 AEH	MPA 70%	74% AEH			71%
		102 EC	MA 15.5%	72% EC			
			Cyclic MPA 5.5%				
			MPA + MA 4%				
		22 IUS (EC)	IUS	68% IUS	IUS N/A	IUS N/A	IUS N/A
Gunderson, 2012 [45]	Systematic Review	391	MPA 49%	All (77%)			117
		111 AEH	MA 25%	AEH 65.8%	AEH 23.2%	AEH 41.2%	
		280 EC	IUS 19%	EC 48.2%	EC 35.4%	EC 34.8%	
			Oral progestins 7%				
			17-hydroxyprogesterone 5.8%				
			OCP, norethisterone, dydrogesterone, oral natural progesterone 5.3%				
			Combination of therapy 8.2%				
Gallos, 2012 [63]	Metanalysis	559	Prog.	EC 76.2%	EC 40.6%	N/A	EC 28.0%
		408 EC	IUS +/− GnRHa	AEH 85.6%	AEH 26%		AEH 26.3%
		151 AEH	HR + Prog/GnRHa				ART 39.4%
			Other				Spont. 14.9%
Koskas, 2013 [44]	Metanalysis	370	MA 20%	Based on Kaplan Meier curve	Based on Kaplan Meier curve	31.60%	N/A
		121 AEH	MPA 54.6%	3 mo: 30.4%	4 mo: 3.6%		
		249 EC	Other 25.4%	6 mo: 72.4%	6 mo: 9.6%		
				12 mo: 78.0%	12 mo: 17.2%		
				18 mo: 80%	18 mo: 26.0%		
				24 mo: 81.4%	24 mo: 29.2%		
Fan, 2017 [46]	Metanalysis	619	Prog 456	76.3% Prog	30.7% Prog	52.1% Prog	N/A
			HR + Prog 73	95.3% HR + Prog	14.1% HR + Prog	47.8% HR + Prog	
			IUS + Prog/GnRHa 90 pt	72.9% IUS + Prog/GnRHa	11.0% IUS + Prog/GnRHa	56.0% IUS + Prog/GnRHa	
Wei, 2017 [48]	Metanalysis	1038	All Prog 1038	71% All Prog	20% All Prog	34% All Prog	20% All Prog
			MPA > 400 mg/day 809	71% MPA > 400 mg/day	33% MPA > 400 mg/day	34% MPA > 400 mg/day	21% MPA > 400 mg/day
			IUS 170	76% IUS	9% IUS	18% IUS	14% IUS
			Prog + IUS 59	87% Prog + IUS	N/a Prog + IUS	40% Prog + IUS	35% Prog + IUS
Guillon, 2019 [58]	Metanalysis	1604	MPA	75% (42–100%)	N/A	N/A	N/A
			MA				
			IUS				
			GnRHa				
			Norethisterone				
			Hydroxyprogesterone caproate				
			Bromocriptine				
			Natural progesterone				
			OCP				
Lucchini, 2021 [7]	Systematic Review	661 EC		79.40%			
			Prog (+GnRHa/M/IUS) 429	77.7% Prog	29.17% Prog	121/429 Prog	81/429 Prog
			HR (+Prog/IUS/GnRHa) 137	90.0% HR	6.93% HR	44/137 HR	35/137 HR
			IUS (+Prog/GnRHa) 95	71.3% IUS	27.03% IUS	18/95 IUS	11/95 IUS
Zhao, 2021 [56]	Metanalysis	446 EC	Oral Prog 279	82% Oral Prog	38% Oral Prog	70% Oral Prog	63% Oral Prog
			HR + Prog/IUS/GnRHa 96	95% HR + Prog/IUS/GnRHa	16% HR + Prog/IUS/GnRHa	84% HR + Prog/IUS/GnRHa	72% HR + Prog/IUS/GnRHa
			IUS +/− Prog/GnRHa 91	69% IUS +/− Prog/GnRHa	30% IUS +/− Prog/GnRHa	48% IUS +/− Prog/GnRHa	36% IUS +/− Prog/GnRHa
Piatek, 2021 [43]	Systematic Review	812	MPA	83%	25.30%	352	246
		231 AEH	MA				
		581 EC	IUS				
			Other Prog				
			HR + Prog/IUS/GnRHa				

Pt: patients; AEH: atypical endometrial hyperplasia; EC: endometrial cancer; MPA: medroxyprogesterone acetate; MA: megestrol acetate; M: metformin; OCP: oral contraceptive pills; GnRHa: gonadotropin-releasing hormone agonist; Prog: progestins; mo: months; CR: complete response; IUS: levonorgestrel-releasing intrauterine system; HR: hysteroscopic resection; ART: artificial reproductive technology; Spont. Spontaneous pregnancy; FU: follow-up; N/A: not applicable.

**Table 2:** Most recent studies on intrauterine systems for endometrial cancer and atypical endometrial hyperplasia.

Study Year	Type of Study	Pt (% or *n*)	Treatment (% or *n*)	CR	Relapse	Pregnancy (% or *n*)	Live Birth (% or *n*)
Kim 2013 [64]	Prospective observational	16 EC	MPA (500 mg/day) + IUS till CR	87.50%	No recurrence in patients with maintenance therapy	3 pt	N/A
			maintenance therapy with OCP or IUS			1 spontaneous	
					2/7 patients that underwent IVF showed recurrent cancer	2 IVF	
Maggiore 2019 [66]	Retrospective	48:	IUS	Overall: 85.4%	Overall: 41.5%	Overall: 73.7%	Overall: 27.3%
		28 AEH		AEH: 89.3%	AEH: 36%	AEH: 54.5%	AEH: 17.9%
		20 EC		EC: 80%	EC: 50%	EC: 100%	EC: 43.8%
Kim 2019 [69]	Multicenter Prospective	44 EC	MPA (500 mg/day) + IUS	37.10%	N/A	N/A	N/A
Xu 2020 [70]	Retrospective	96:	HR + IUS (32)	HR + IUS 81.0%	HR + IUS 19.2%	N/A	N/A
		59 AEH	HR + MA (160–320 mg/die) (32)	HR + MA (160–320 mg/die) 90.6%	HR + MA (160–320 mg/die) 10.3%		
		37 EC	HR + IUS + MA (160–320 mg/die) (32)	HR + IUS + MA (160–320 mg/die) 87.5%	HR + IUS + MA (160–320 mg/die) 10.7%		
Janda 2021 [67]	Randomized clinical trial	154	IUS (35)	IUS 61%	IUS 9%	N/A	N/A
		42% EAH	IUS + weight loss intervention (36)	IUS + weight loss intervention 67%	IUS + weight loss intervention 3%		
		58% EC	IUS + metformin (47)	IUS + metformin 57%	IUS + metformin 17%		
Westin 2021 [65]	Prospective	57:	IUS	AEH + EC: 79%	AEH + EC: 9.5%	N/A	N/A
		36 AEH		AEH: 91%			
		21 EC		EC: 54%			
Novikova2021 [68]	Retrospective	418:	AEH: 1. IUS + 2 D & C 124 pt 2. IUS + GnRHa +3 D & C 20 pt 3. IUS + 3 D & C 45 pt 4. MPA + 3 D & C 39 pt	Overall: AEH: 96% EC: 88%	AEH: 26%	68%	42%
		228 AEH		AEH: 1. IUS + 2 D & C 98% 2. IUS + GnRHa + 3 D & C 95% 3. IUS + 3 D & C 100% 4. MPA + 3 D & C 87%	EC: 36%	38% ART	
		190 EC	EC: 1. IUS + GnRHa +2 D & C 83 pt 2. IUS + GnRHa + MPA + 3 D & C 24 pt 3. IUS + GnRHa +3 D & C 56 pt 4. MPA + 3 D & C 27 pt	EC: 1. IUS + GnRHa + 2 D & C 89% 2. IUS+ GnRHa + MPA + 3 D & C 71% 3. IUS + GnRHa + 3 D & C 96% 4. MPA + 3 D & C 81%			
Piatek 2021 [43]	Case series	30:	IUS 20%	Overall: 70% IUS: 83.3% Low dose Prog: 60% High dose Prog: 71.4%	IUS: 20%	4 pt	3 pt
		10 AEH			Low dose Prog: 17%		
		20 EC	Prog 80%: Low dose Prog: (MA 80 mg daily; MPA 150 mg or 500 mg^2^/week) High dose Prog: (≥160 mg MA)	Probability of CR using Kaplan–Meier: IUS: 83.3% Low dose Prog: 55.6% High dose Prog: 73.3%	High dose Prog: 40%		

Pt: patients; AEH: atypical endometrial hyperplasia; EC: endometrial cancer; BMI: body mass index; MPA: medroxyprogesterone acetate; MA: megestrol acetate; M: metformin; OCP: oral contraceptive pills; GnRHa: gonadotropin-releasing hormone agonist; Prog: progestins; mo: months; CR: complete response; IUS: levonorgestrel-releasing intrauterine system; HR: hysteroscopic resection; IVF: in vitro fertilization; D&C: dilation and curettage; SD: stable disease; FU: follow-up; N/A: not applicable; PD: Progressive Disease.

**Table 3:** Most recent studies on metformin therapy for endometrial cancer and atypical endometrial hyperplasia.

Study, Year	Type of Study	Patients (*n*)	Treatment (*n*)	Treatment Dosage	CR	Relapse	Pregnancy	Live Birth
Mitsuhashi, 2019 [75]	Retrospective	63 total	Prog + M	MPA 400 mg + Metformin 750–2250 mg daily	97% Prog + M	13.1% Prog + M	61%	45%
		21 AEH			95% EC			
		42 EC			100% AEH			
		23	Prog		87% Prog	50% Prog		
Acosta-Torres, 2020 [20]	Retrospective	92	Prog 58	MA 80–160 mg daily or	69%	16%	N/A	17%
		33 AEH + 25 EC	Prog + M 34	MPA 10–40 mg daily or	69% Prog	20% Prog		24% Prog
		21 AEH + 13 EC		Prometrium 400 mg daily or	68% Prog + M	9% Prog + M		6% Prog + M
				LNG-IUS 52 mg				
				Metformin 500–1000 mg daily				
Yang, 2020 [78]	Randomized control trial	150		MA 160 mg daily				
		62 AEH + 12 EC	Prog 74	Metformin 500 mg three times a day	68.2% Prog	9.1% Prog	48.4% Prog	41.9% Prog
		61 AEH + 15 EC	Prog + M 76		74.3% Prog + M	10.1% Prog + M	51.8% Prog + M	21.6% Prog + M
Matsuo, 2020 [77]	Retrospective	245 AEH	Prog 140	Prog; IUS;	Prog 27.8%	N/A	N/A	N/A
			Prog + M 36	Metformin	Prog + M 23.1%			
			IUS 54	Dosages not specified	IUS 58.9%			
			IUS + M 15		IUS + M 86.7%			

AEH: atypical endometrial hyperplasia; EC: endometrial cancer; MPA: medroxyprogesterone acetate; MA: megestrol acetate; M: metformin; IUS: levonorgestrel-releasing intrauterine system; Prog: progestins; mo: months; w: weeks; CR: complete response.

## Data Availability

Not applicable.
